# Supplementary material for: Understanding the extent of and limitations to conscientious objection to abortion by health care practitioners: A hermeneutic study
Source: PLoS One. 2024 Feb 23;19(2):e0297170. doi: 10.1371/journal.pone.0297170 (PMC10889651; doi:10.1371/journal.pone.0297170)
Supplement: Attachment — Submitted filename: PONE response.docx [file pone.0297170.s001.docx]

Dear editor and reviewers

Thank you so much for your sharp eyes and critical comments. On behalf of the team, I enclose a response to your comments as well as the revised manuscript.

Kind regards

Valerie

| 1. Please ensure that your manuscript meets PLOS ONE's style requirements, including those for file naming. | Thank you, done |
| --- | --- |
| 2. We noticed you have some minor occurrence of overlapping text with the following previous publication(s), which needs to be addressed. In your revision ensure you cite all your sources (including your own works), and quote or rephrase any duplicated text outside the methods section. Further consideration is dependent on these concerns being addressed. | We’ve been through this and cannot find anything that has not been cited including team members’ previous publications. Please advise is we’ve overlooked something. |
| 3. We note that the grant information you provided in the ‘Funding Information’ and ‘Financial Disclosure’ sections do not match. When you resubmit, please ensure that you provide the correct grant numbers for the awards you received for your study in the ‘Funding Information’ section. | Sorry, sorted. The mistake came about because additional funding was received during the pandemic. |
| 3. Have the authors made all data underlying the findings in their manuscript fully available?  The [PLOS Data policy](https://ddec1-0-en-ctp.trendmicro.com:443/wis/clicktime/v1/query?url=http%3a%2f%2fwww.plosone.org%2fstatic%2fpolicies.action%23sharing&umid=396fdfda-2ba2-44f8-8ab6-8053d1f379e8&auth=6b639a990a359ff1d6cc8761081d57748ce3c81e-9fdff9ebe3314290b55a17e5782b1604d422286a) requires authors to make all data underlying the findings described in their manuscript fully available without restriction, with rare exception (please refer to the Data Availability Statement in the manuscript PDF file). The data should be provided as part of the manuscript or its supporting information, or deposited to a public repository. For example, in addition to summary statistics, the data points behind means, medians and variance measures should be available. If there are restrictions on publicly sharing data—e.g. participant privacy or use of data from a third party—those must be specified. |  |
| Reviewer #1: Yes |  |
| Reviewer #2: No | All data is available on the link listed. We fail to see why “partly” has been written here. |
| Reviewer #1: This is very interesting empirical research that addresses an important topic. However, the paper reads as a rough first draft rather than a final, polished article. | Sorry, that is how you feel. A lot of consultation and work went into the presentation. It was definitely NOT a rough first draft. However, there is always room for improvement and we have gone through it again making subtle and not so subtle changes. |
| While methodology is important, I don’t think that the detailed description of the methodology is necessary. It should not take until page 8 to get to the substance of the article. I recommend condensing the content of the article up to page 8 which is essentially an overly detailed description of the methodology. | This was difficult because the other reviewer wanted more detail. In fact the results began on page 5 (commencing on page 2) not page 8 of the Pdf submitted. We have, however, trimmed the methodology back a bit. |
| While the researchers have clearly engaged in a lot of work figuring out what are the themes of the data, these themes are presented as a series of quotes from the interviews and are lacking in analysis. Presenting the quotes is step 1, but the authors then need to go on to discuss and analyse those quotes and how they relate to the identified theme. I think it would make sense to restructure the article so that the discussion is included in the analysis of the themes, rather than being presented separately. Otherwise the setting out of the themes is just a series of quotes. Alternatively, the authors may wish to present a more traditional version of a results section followed by discussion. | Yes, we agree. We have integrated the findings and discussion as it seems to read better that way. |
| The way that the views of the authors is presented is problematic. On the one hand, the authors frame the research as being about 2 questions: 1. What do health professionals understand as constituting ‘participation in abortion’? 2. On what grounds should health professionals be permitted to withdraw from provision of abortion services? But the article does not clearly address these two questions. | Again, food for thought. You are right. We have amended our conclusions to reflect this. |
| Further, the authors seem to adopt a position on whether conscientious objection should be allowed and in what circumstances. It is not in and of itself problematic for the authors to adopt a position, but they don’t admit to doing so in the article – their position is presented as fact rather than opinion and is not clearly based on the data. | We can’t see where we have presented our own positions. In fact we all held different ones. We have updated the detailed section on our own pre-understandings. |
| Reviewer #2: Overall, the manuscript contributes much-needed empirical data on the topic of health practitioners' CO to abortion. More background context is needed to understand the results of the study, and the authors should demonstrate more rigour in places (particularly given the ethically controversial nature of abortion, and CO).  Background: More context is needed about the current UK legal framework as it relates to CO to abortion. Is CO to abortion protected in legislation? If so, which health practitioners does the clause apply to? What are the legal requirements for claiming CO? And what are the objecting practitioner’s obligations e.g. declaring the CO to the patient, referring on etc? If CO is legally protected, then this would surely have implications for whether health services are required to accommodate it?  Also, further detail is needed about the context of abortion provision in the UK. For example, are abortions all publicly funded? Or does the private sector play a role? What is the pathway for having an abortion e.g. explanation of the form requiring two signatures?  p. 4:  “Little has been written about other health professionals’ views or experiences of conscientious objection , despite recent legal challenges to laws in Scotland, Croatia and Sweden by midwives who objected to abortion provision on grounds of conscience”  There are other empirical studies that focus on or include non-doctor CO to abortion such as:  • Awonoor-Williams et al Exploring conscientious objection to abortion among health providers in Ghana. International Perspectives on Sexual and Reproductive Health 2020 Vol. 46 Pages 51-59 • Davidson et al. Religion and conscientious objection: A survey of pharmacists’ willingness to dispense medications. Social Science&Medicine71(2010)161e165 • Harries et al 2014 Conscientious objection and its impact on abortion service provision in South Africa: a qualitative study. Harries et al. Reproductive Health 2014, 11:16 <https://ddec1-0-en-ctp.trendmicro.com:443/wis/clicktime/v1/query?url=http%3a%2f%2fwww.reproductive%2dhealth%2djournal.com%2fcontent%2f11%2f1%2f16&umid=396fdfda-2ba2-44f8-8ab6-8053d1f379e8&auth=6b639a990a359ff1d6cc8761081d57748ce3c81e-2664dc5e8bc258c533ca3f73418ec6a8cb368adf> • Ko et al et al An ethical issue: nurses’ conscientious objection regarding induced abortion in South Korea. • Lee et al. Mifepristone (RU486) in Australian pharmacies: the ethical and practical challenges. Contraception 91 (2015) 25–30.  . | See comments above to the other reviewer. We have added some and removed some and we hope it is now satisfactory to you both. |
| Methods:  p. 5: “Finally, six months after commencing data analysis, we came together and explored how our pre and altered understandings contributed to and influenced our project, recognising that as Gadamer notes, it is only by consciously assimilating preunderstandings that we can avoid “the tyranny of hidden prejudices that make us deaf to the language that speaks to us in tradition” (Gadamer, 2006, p. 239).”  It’s good you have formally reflected on your understandings of CO and how they have contributed and influenced the project. Please provide some description about these understandings and how they influenced your data collection and analysis. | See comments to other reviewer. I think we’ve now addressed this. |
| Recruitment and participants: Good sample size with both objectors and non-objectors. Range of health practitioners included. | Thank you |
| Data collection: Please provide the interview guide as an appendix or supplemental material.  Tables 1 and 2 are not integrated with the main body of the text. | Done, thanks. |
| How did you ensure rigour in developing the continua? | Sentence added, thank you. |
| P. 13 – information about referral requirements should be part of the Background  Referencing software needs attention – incorrect bracketing of in-text citations throughout | Both these done |
| Conclusions: “Our research represents the largest qualitative empirical study and the first in Europe on the subject of health professionals’ conscientious objection to abortion.”  Agree it is a large qualitative, empirical study. But I’m not sure that it’s the first in Europe? What about these?  • De Zordo (2018) From women’s ‘irresponsibility’ to foetal ‘patienthood’: Obstetricians-gynaecologists’ perspectives on abortion and its stigmatisation in Italy and Cataluña. GLOBAL PUBLIC HEALTH, 2018 VOL. 13, NO. 6, 711–723 • Nordberg (2014) Conscientious objection to referrals for abortion:pragmatic solution or threat to women’s rights? Medical Ethics 2014, 15:15 <https://ddec1-0-en-ctp.trendmicro.com:443/wis/clicktime/v1/query?url=http%3a%2f%2fwww.biomedcentral.com%2f1472%2d6939%2f15%2f15&umid=396fdfda-2ba2-44f8-8ab6-8053d1f379e8&auth=6b639a990a359ff1d6cc8761081d57748ce3c81e-7030e6466c730d252a18813d7ba0d805b0ff6b11> • Toro-Flores (2019) Opinions of nurses regarding conscientious objection. Nursing Ethics, 2019, Vol. 26(4) 1027–1038 | You are right. Sentence has been amended.  Thanks for the references. Most of them we already had but the de Zordo was new and really interesting. It’s a shame it was only on drs! |
| Conclusion Respecting self and others shows that participants who were objectors were accepted by their colleagues and accommodated without detriment to the service, and that in larger hospitals…  The result below sounds like a detriment to the service?  A doctor described the hierarchy in a pre-abortion clinic: whilst I’m seeing the next one, the nurses would have to try and walk around and find somebody that would do a second signature. And, again, if we’re thin on the ground of people to find, and those people that are in are… We had nobody to sign the document (doctor, non-objector). P. 11  “ | You have a good point. Thank you. Amended. We are going to create another article specifically about nurses in this regard. |
| Our study was carried out in two large urban areas with specialised women’s reproductive health services.” This context would be useful in the methods section. Also, perhaps this could be a reason that difficulties with abortion access stemming from CO did not feature in this study? | Possibly but it’s these hospitals that offer the range of abortion services. |
| Conclusion could be more concise | Thank you. Done. |
