# Supplementary material for: Understanding the extent of and limitations to conscientious objection to abortion by health care practitioners: A hermeneutic study
Source: PLoS One. 2024 Feb 23;19(2):e0297170. doi: 10.1371/journal.pone.0297170 (PMC10889651; doi:10.1371/journal.pone.0297170)
Supplement: Attachment — Submitted filename: Reviewers response.docx [file pone.0297170.s002.docx]

| Reviewer #2: Revisions have improved the manuscript. Given members of the author team held different personal positions on CO  I think this should be stated explicitly in the paper. It is a strength of the author team and of the paper. | Thank you, we have strengthened this. It’s difficult at times to differentiate what is necessary to say and what should not be said but I certainly do not want to be accused by readers or being a “CO club”! |
| --- | --- |
| Reviewer #3: The authors deal with a particularly hot topic and very much addressed in literature from various points of view. Therefore it is inevitable that my opinions and comments consider the originality of the content.  Firstly in my opinion there is one key point missing from the introduction namely an argument on bioethical issues of conscientious objection. On which rights does conscientious objection rest? what does it aim to protect? Why is this right granted even in circumstances where it concerns the psychophysical health of patients?  I find that such a premise is fundamental to give substance to the introduction which at present is too superficial and is far from taking stock of the real issue. In addressing issues of this nature we should start from a solid bioethical argument indispensable to be able to effectively argue the answers provided by health professionals interviewed.  In fact even on such occasions the authors' arguments are rather hasty. | It is indeed a hot topic and thank you for the critique. We all appreciate it.  We have each had a go at this and our ethicist on the authors has particularly strengthened it. The simple answer perhaps is in your own question re the health of patients. I think the objectors felt that they had two patients….one who couldn’t speak for themselves. However, that’s just FYI as we could go down a whole different pathway. It’s really great that we have an ethicist on the team who is able to move into empirical research and we hope this article will have some “punch” with clinical practitioners. |
| Yet the results of the questionnaires are quite interesting so it would be a shame if they were lost. | Just a bit of puzzlement here…we didn’t use questionnaires. |
| I invite the authors to review these aspects trying to unbalance themselves a little more in the discussions and giving a more critical imprint to the article also considering the great experience of the authors on the subject. | Thank you, we’ve tried our best! |
| I hope that authors can find my comment motivating as I do myself with other reviewers when I am in their position. | Indeed we do and it would be good to be able to have more debate in the future. Thank you for coming on board as a last minute substitution in the reviewers. |
